# Underwater Repair of Concrete Elements with TRC Grouting System

**DOI:** 10.3390/ma15134469

**Published:** 2022-06-24

**Authors:** Hyeong-Yeol Kim, Young-Jun You, Gum-Sung Ryu

**Affiliations:** Structural Engineering Department, Korea Institute of Civil Engineering and Building Technology (KICT), Goyang-Si 10223, Korea; hykim1@kict.re.kr (H.-Y.K.); ryu0505@kict.re.kr (G.-S.R.)

**Keywords:** carbon textile, cementitious grout, concrete structure, bond test, textile-reinforced concrete (TRC), structural testing, underwater repair

## Abstract

The repair of underwater concrete structures is usually difficult work, requiring specialized materials and installation systems. This paper presents a carbon-textile-reinforced concrete (TRC) grouting system for underwater repair of concrete structures. One multi-purpose grout and two types of underwater grouts were considered in this study, and the bond performance between the substrate and grout was evaluated by a bi-surface shear test with cubic specimens. The bond strength of the repair material is greatly affected by the casting and curing conditions. When the multi-purpose grout is used, the average bond strength of the specimens cast and cured in dry conditions is only 22% of the specimens cast and cured in underwater conditions. On the other hand, the maximum difference in bond strength is, at most, 15.8% when non-dispersive, anti-washout grouts are used. Two types of installation methods were proposed and four full-scale RC slab specimens were repaired with the TRC grouting method, two for each installation method. Regardless of the installation method, the load levels that causes concrete cracking, steel yield, and the failure of specimens repaired with the TRC grouting system are at least 37.5%, 16.6%, and 21.7% greater than those of the unrepaired specimen, respectively. The test results further indicate that the influence of the grouting materials on the ultimate load-carrying capacity of the specimens repaired with the TRC grouting system is insignificant, and the maximum difference is, at most, 4%.

## 1. Introduction

Civil infrastructures including bridges, hydraulic structures, and waterfront structures are often constructed in tidal zones or underwater environments. These structures are generally constructed with reinforced concrete (RC) and, if they are located in an underwater environment, are referred to as underwater concrete structures [1]. Underwater concrete structures can be damaged by abrasive erosion or impact. If damaged and deteriorated sections of concrete structures are not properly repaired, the service life decreases, and the life-cycle maintenance cost dramatically increases. Therefore, damaged or deteriorated underwater concrete structures should be repaired with the appropriate repair materials and systems.

The repair of underwater concrete structures is usually difficult work, requiring specialized materials and installation systems [1]. Tremie concrete and pumped concrete are commonly used for underwater concrete repair of a larger damaged area [2]. On the other hand, for a smaller damaged area, forms or jackets are installed over the section to be repaired, and then cementitious grouts are pumped to fill voids [1,3]. Grouts can be filled through gravity flow if the grout has a high workability. In a practical application, textile grid or fabric are also used to prevent the grout from coming out of the voids [4].

Removable forms and jackets, such as plywood forms and steel jackets, are commonly used for grouting repair of underwater concrete structures. In recent years, fiber-reinforced polymer (FRP) composites were used as stay-in-place (SIP) jackets for the underwater repair of concrete elements [5,6]. If SIP FRP jackets are used, significant time and resources can be saved during the repair work, when compared to the use of removable jackets [7]. Although the SIP FRP jacket has many advantages over the conventional steel jackets, full composite action between the SIP jacket and grout cannot be obtained, because the bond performance between the FRP jacket and grout is generally weak.

In recent years, textile-reinforced concrete (TRC), consisting of a textile grid and cement-based matrix, was widely used for the repair and strengthening of deteriorated or structurally deficient concrete elements under various environmental conditions [8,9,10,11]. RC beams [9,12] and RC slabs [13,14] were successfully strengthened with a cast-in-place (CIP) TRC system. Precast TRC panels were used in new construction [15,16], and in strengthening existing structures [17]. A lack of design guidelines and testing methods for TRC systems was one shortcoming of the TRC systems over the conventional system in earlier applications. However, the design guidelines and testing methods for the TRC system have been successfully established [18,19]. The state of the art development and application of TRC for concrete elements can be found in the literature [20].

A research group in KICT developed a carbon-textile-reinforced concrete (TRC) panel, as not only an SIP formwork for the RC slab-type element of an open-type wharf structure during construction, but also as a protective layer during service [21]. More recently, a TRC panel grouting system was proposed for flexural strengthening of RC slab-type elements [22,23]. In these studies, TRC panels were fabricated with a carbon textile grid, and cementitious mortar and the prefabricated TRC panels were externally bonded to existing RC slabs by grouting. The results of these studies indicate that the TRC panel grouting system can be utilized as an effective repair or strengthening method for RC elements with a limited working space, or with difficult accessibility.

A thorough search of the relevant literature on TRC indicated that neither the development nor the application of TRC in an underwater environment has been reported. This paper proposes a TRC grouting system and installation method for the underwater repair of concrete structures. The proposed TRC grouting system consists of a carbon textile grid and cementitious grout. The objectives of this paper are to evaluate proposed underwater installation methods for a TRC grouting system, and to experimentally validate the structural performance of the RC elements repaired with the proposed installation methods. A carbon textile grid and three different grouts (one multi-purpose grout and two underwater grouts) were considered for underwater installation of the TRC grouting system.

A total of 18 cubic specimens with a bi-surface (interface) between the substrate and repair materials were cast underwater and tested in shear to examine the bond performance of the material interface. To validate the proposed installation method of the TRC grouting system, five 2000.0 mm long RC slabs were fabricated, and two of the RC slabs were repaired with each installation method in an underwater environment. The RC slabs repaired with the TRC grouting system were tested in flexure, and the structural performance of the specimens was compared with that of an unrepaired RC slab and the analytical solutions.

## 2. Material Tests

### 2.1. Materials

Two types of substrate materials (concrete and mortar) and three types of cementitious grouts were considered in the bonding test for the substrate with the repair material. For the fabrication of the substrate, a ready-mixed concrete and a commercially available mortar were used, and the mix composition of concrete and mortar are provided in Table 1.

A Portland cement-based non-shrink grout and two types of cementitious grouts for underwater application were selected for repair materials for the bonding test (Table 2). G1 is a multi-purpose grout for concrete repair that is normally used in a dry condition. The mix composition of G1 suggested by the manufacturer is summarized in Table 3. G1 was also used for TRC strengthening of a RC element in a previous study [25]. On the other hand, the non-dispersive, anti-washout grouts for underwater application (G2 and G3) are mixed with special additives, but the mix composition is unknown. Note that the design strength of G3 is 1.67 times greater than that of G2.

### 2.2. Test Specimens

A bi-surface direct shear test [26] with a cubic specimen (150.0 mm × 150.0 mm × 150.0 mm) was used to evaluate the bond strength between the repair materials and substrate. In the bi-surface shear test, the repair material is cast to a height of 50.0 mm over a 100.0 mm thick substrate.

**Figure 1:**
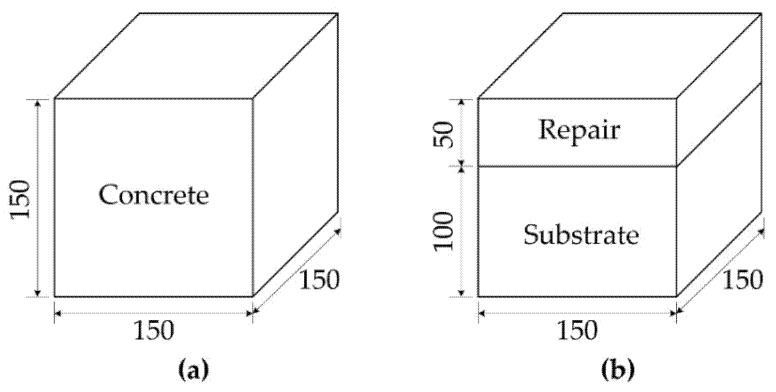
Cubic specimens for bi-surface direct shear test: (**a**) concrete (Type A); (**b**) concrete with repair material (Type B) (unit: mm).

Table 4 summarizes the characteristics of the three groups of specimens tested for the bi-surface shear test. The design variables considered in the test are the type of substrate and repair materials, two material interfaces, and the casting condition for the repair materials. Type A specimen (Figure 1a) was cast with concrete only and, hence, STC specimen is a control specimen to identify the direct shear strength of the concrete substrate. On the other hand, for the Type B specimen (Figure 1b), a substrate with a height of 100.0 mm was cast first, and cured for seven days, and then 50.0 mm thick repair material was cast on the substrate. Therefore, Type B specimen has either a concrete–grout interface, or a mortar–grout interface.

**Figure 2:**
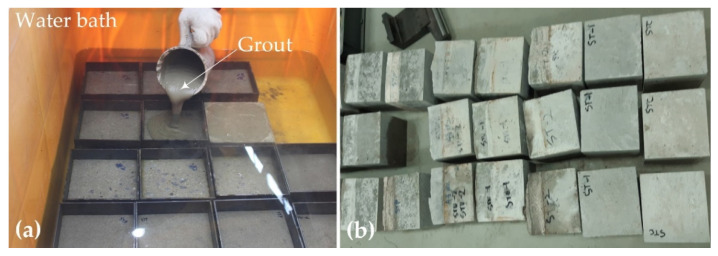
(**a**) Underwater grouting process in a water bath; (**b**) water-bath-cured test specimens.

Figure 2a shows the underwater grouting process of the repair material through gravity flow. The specimens were cast in a water bath, and then cured in water for 28 days. Figure 2b shows the water-bath-cured specimens after curing.

### 2.3. Results of Bond Strength Tests

The compressive strengths of the substrate and repair materials were evaluated by the compressive strength testing method. The compressive strengths of concrete and mortar are identified as 32.9 MPa and 36.5 MPa, respectively. On the other hand, the compressive strength of G1, G2, and G3 are identified as 53.6 MPa, 46.7 MPa, and 27.4 MPa, respectively.

**Figure 3:**
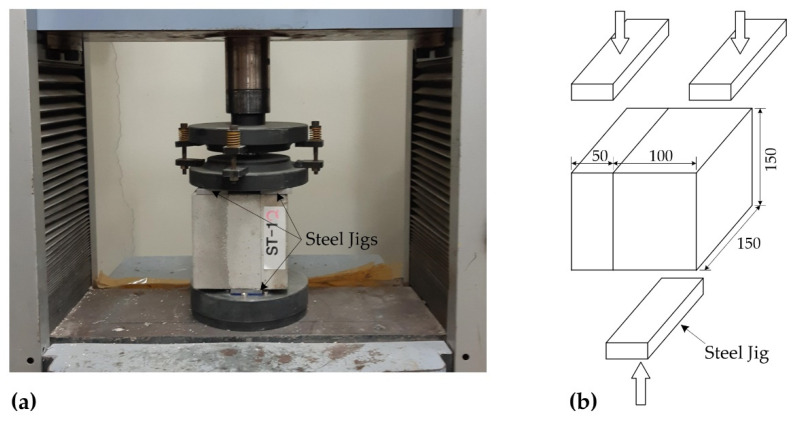
(**a**) Set-up for bi-surface direct shear test; (**b**) loading scheme with steel jigs (unit: mm).

Figure 3a shows the set-up for the bi-surface direct shear test. Vertical monotonic loading was applied to the specimens by three steel jigs, two on the top and one on the bottom (Figure 3b).

**Figure 4:**
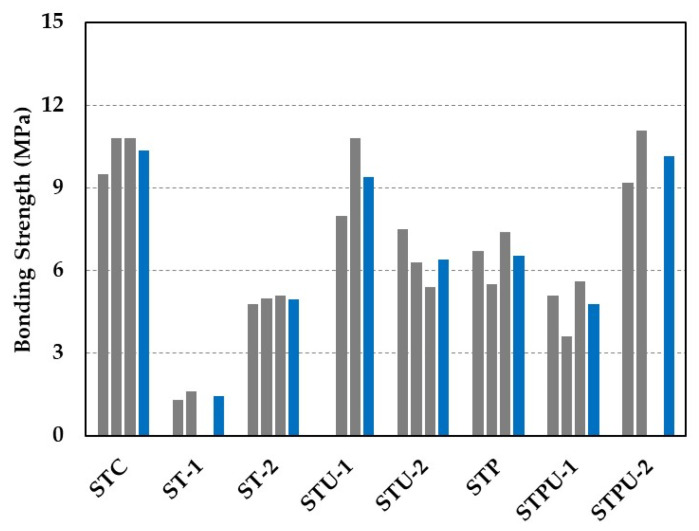
Direct shear test results for all sets of specimens (blue color = average value of tests).

The bond strength (*σ*) of the specimens is calculated by [26]

(1) $$\sigma =\frac{P}{A}$$

where P = maximum load at failure (N), and A = area of the bond plane (mm^2^). Figure 4 shows the bond strength for all sets of the specimens, and average values of the test results are summarized in Table 5.

The average bond strength of concrete without repair material (STC series) is found to be 10.4 MPa. On the other hand, the average bond strength of the concrete substrate with multi-purpose grout (G1) cast and cured under dry and underwater conditions is 1.5 MPa and 5.0 MPa, respectively. The bond failure occurs at the concrete–grout interface for the ST-1 and ST-2 series specimens. The bond strength varies greatly depending on the casting and curing conditions. Although the same grout (G1) is used as a repair material, the average bond strength of the ST-1 series specimen (dry casting) is only 30% of that of the ST-2 series specimen (underwater casting and curing). Note that the grout was cast without pre-wetting of the concrete substrate for ST-1 series specimens. Therefore, mixing water from the grout might be absorbed into the concrete substrate, and this results in an adverse effect on the bond strength of the concrete–grout interface. A research group [27] also conducted a bond test for a concrete–grout interface, and the test results indicate that the bond performance of the interface is significantly affected by moisture in the concrete substrate.

The average bond strength of the STU-1 and STU-2 series specimens (the concrete with non-dispersive, anti-washout grouts) is evaluated as 9.4 MPa and 6.4 MPa, respectively, and bond failure also occurs at the concrete–grout interface for these specimens. Although the higher compressive strength grout results in higher bond strength, the maximum difference is 31.9%. Therefore, the bond strength of specimens with the concrete–grout is greater than 5.0 MPa, regardless of the grout type used when they are cast and cured in the underwater environment.

**Figure 5:**
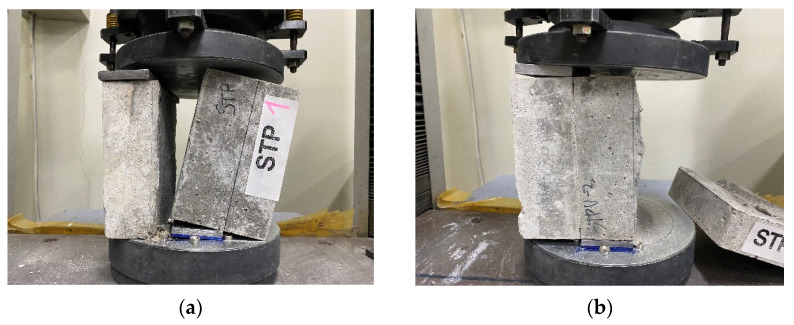
Failure modes of specimens: (**a**) interfacial failure (STP); (**b**) substrate failure (STPU-2).

The average bond strength of the STP specimen (the mortar with multi-purpose grout) is identified as 6.5 MPa. As shown in Figure 5a, bond failure occurs at the mortar–grout interface.

The average bond strength of the STUP-1 and STUP-2 specimens (the mortar with non-dispersive, anti-washout grouts) is evaluated as 4.8 MPa and 10.2 MPa, respectively. Bond failure occurs at the mortar–grout interface for the STPU-1 specimens. On the other hand, two of the STPU-2 series specimens fail at the mortar–grout interface, while one specimen (STPU-2-2, Figure 5b) experiences shear failure of the mortar substrate. Note that the tested value for the STPU-2-2 specimen is 11.1 MPa, and this value is considered to be the shear strength of the mortar, rather than the bond strength between the mortar and grout.

## 3. Structural Test for RC Slabs Repaired with TRC Grouting System

### 3.1. TRC Grouting System for Damaged Concrete Structures

**Figure 6:**
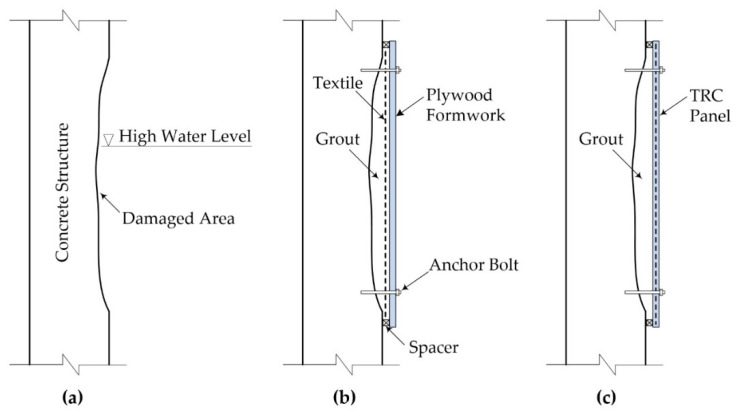
(**a**) Damaged concrete structure; (**b**) TRC grouting system with removable formwork; (**c**) TRC grouting system with TRC panel.

Figure 6a depicts a damaged concrete structure in a tidal zone. In this study, two installation methods for the TRC grouting system are proposed for repair of the damaged section: the TRC grouting system cast with removable formwork (Figure 6b); and the TRC grouting system cast with TRC panels (Figure 6c). The deteriorated concrete cover (the deteriorated concrete section) should be completely removed prior to carrying out the TRC grouting system repair. The grout is pumped to fill the section to be repaired, and then cracks in the existing concrete are filled with the grout.

### 3.2. Fabrication of Full-Scale RC Specimens

Full-scale RC structural elements were repaired with the proposed TRC grouting system in a laboratory, and tested in flexure to validate the effectiveness of the proposed system in repair work. Four 2000.0 mm long and 500.0 mm wide RC slab-type elements were designed and fabricated to represent the full-scale RC element to be repaired with the proposed system.

**Figure 7:**
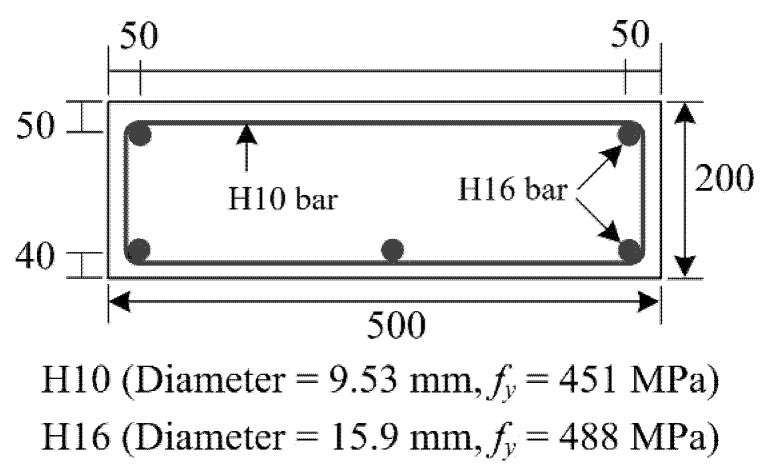
Cross-sectional dimensions and reinforcement details (unit: mm).

Figure 7 shows the cross-sectional dimensions and steel arrangement of the RC element. In this experiment, the RC element is assumed to be located in an underwater environment and undergoes steel corrosion, such that repair work with additional placement of reinforcement is required to recover loss of the steel cross-section in the existing element. In this study, the additional reinforcement is provided by the TRC grouting system. For this reason, the original RC element was designed as an under-reinforced element. Note that the mix composition and the design strength of the concrete are the same as those used in the bonding test (Table 1). The diameter and the yield strength of steel reinforcement (*f_y_*) are provided in Figure 7. Transverse steel reinforcement (H10) was spaced at 125 mm in the longitudinal direction of the RC element, to prevent shear cracking of the specimen during the test.

### 3.3. RC Slabs Repaired with TRC Grouting System

**Figure 8:**
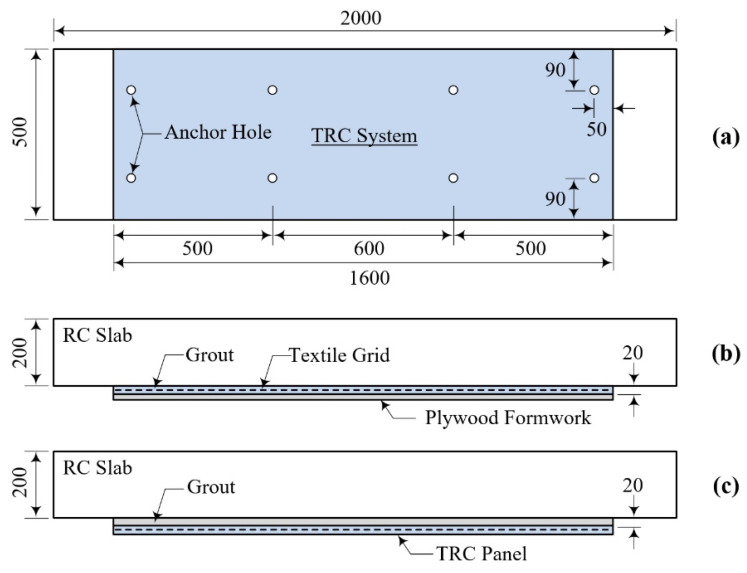
Repair plan for RC element with TRC grouting system: (**a**) repaired section; (**b**) CUW method; (**c**) PUW method (unit: mm).

In the proposed method for underwater repair of RC elements, two types of installation methods for the TRC grouting system are used: a CIP TRC grouting system (CUW method) with removable formwork, and a TRC grouting system with a prefabricated TRC panel (PUW method). Figure 8a depicts the full-scale RC element repaired with the TRC grouting system. Furthermore, Figure 8b,c illustrate side views of the full-scale RC element repaired with the CUW and PUW methods, respectively.

Table 6 summarizes characteristics of the underwater-repaired RC specimens to be tested in flexure. Note that the RC specimen is a control specimen (Figure 7), without the TRC grouting system. In previous studies [25,28], RC slab specimens were strengthened by the CIP TRC grouting system with a multi-purpose grout (G1, Table 3). Note that, except for the grout, the dimensions and materials used for the specimens in the previous studies [25,28] are identical to those of the CUW series specimens tested in this study.

**Figure 9:**
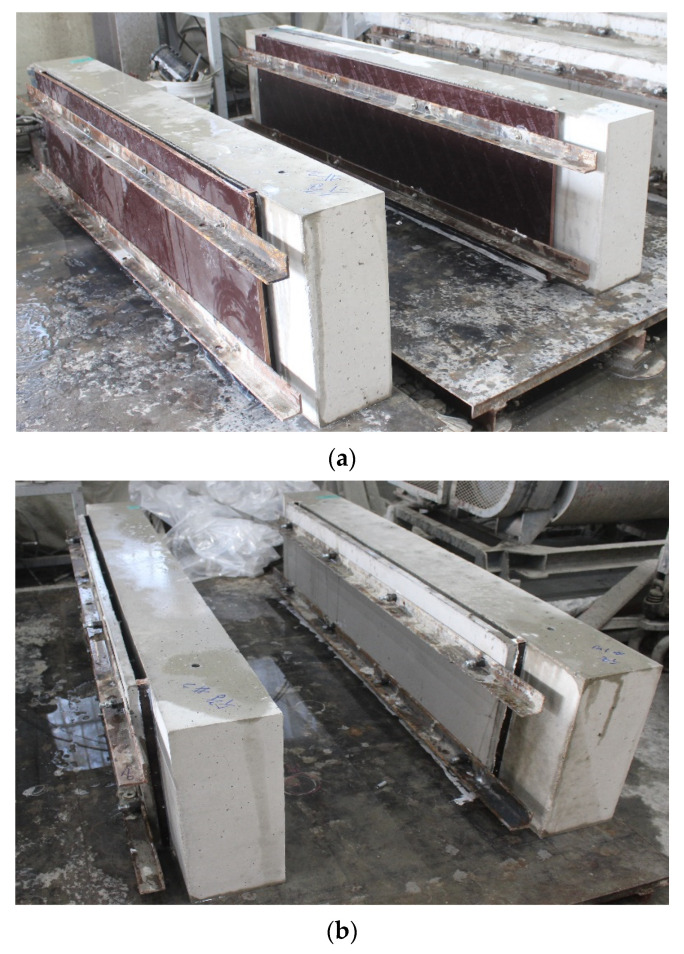
Fabrication of RC slab specimens with TRC grouting system: (**a**) CUW series; (**b**) PUW series.

Figure 9 shows the plywood formwork and the TRC panel assembled to the RC elements by eight stainless steel anchor bolts (diameter = 5 mm) and L-shaped steel angles. A single ply carbon textile was also installed for the CIP TRC system (CUW series specimens). On the other hand, a single ply carbon textile and the mortar outlined in Table 1 were used to fabricate a 20.0 mm thick TRC panel. Table 7 summarizes the material properties of the carbon textile grid.

**Figure 10:**
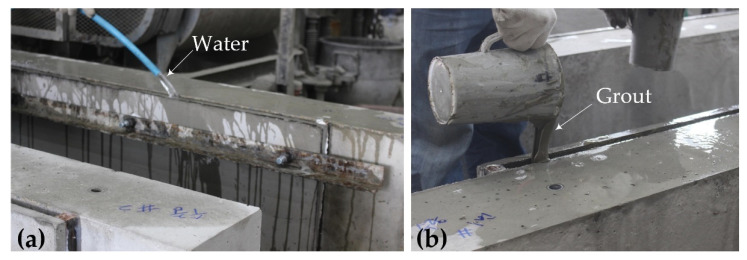
TRC grouting process: (**a**) Water filling; (**b**) grouting.

As shown in Figure 10a, the void between the plywood formwork or the TRC panel and the RC elements was filled with water, and maintained in water for 4 h to simulate the underwater environment prior to the grouting process. Note that a much longer water contact period for the specimen should be considered to simulate the conditions of submerged concrete structures in a realistic manner. The non-dispersive, anti-washout grouts (G2 and G3, Table 2) were filled through gravity flow in the voids of RC specimens for underwater repair (Figure 10b).

**Figure 11:**
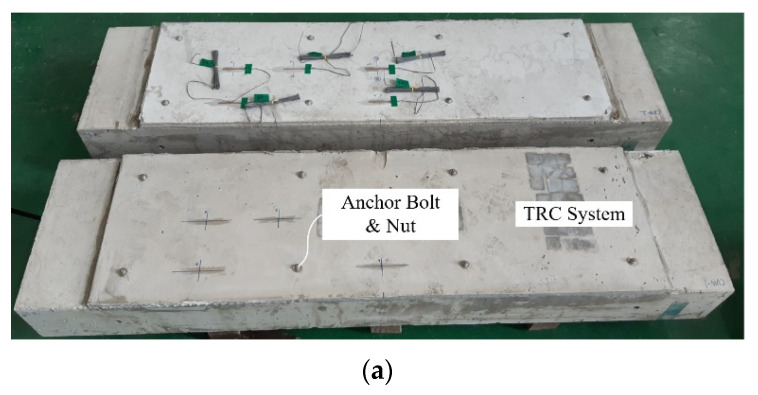

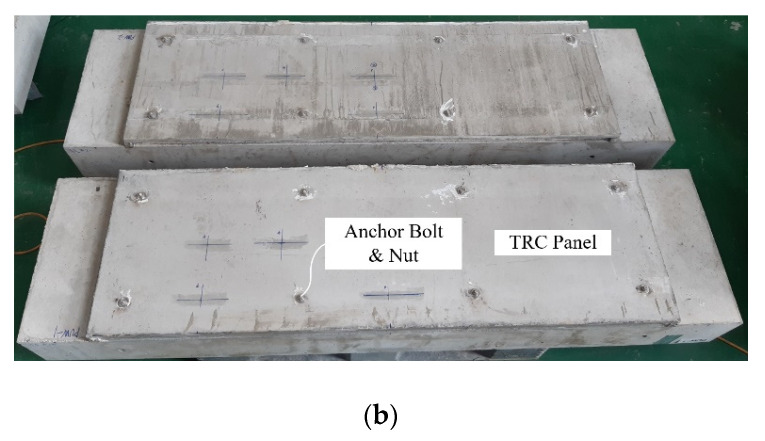
TRC system repaired RC specimens: (**a**) CUW series; (**b**) PUW series.

Figure 11a,b show the RC elements repaired with the CUW and PUW methods, respectively. The anchor bolts used to assemble the plywood formwork and TRC panel to the RC elements remained after curing the specimens, and were tightened with eight sets of stainless steel washers and nuts. The anchor studs partially resist the debonding stress between the TRC grouting system and the RC element.

### 3.4. Load-Displacement Behavior of RC Slab Repaired with TRC Grouting System

**Figure 12:**
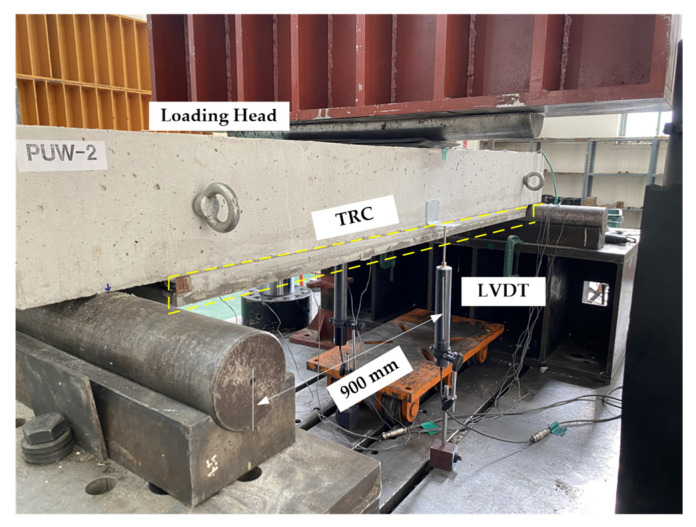
Flexural test set-up and instrumentation.

**Figure 13:**
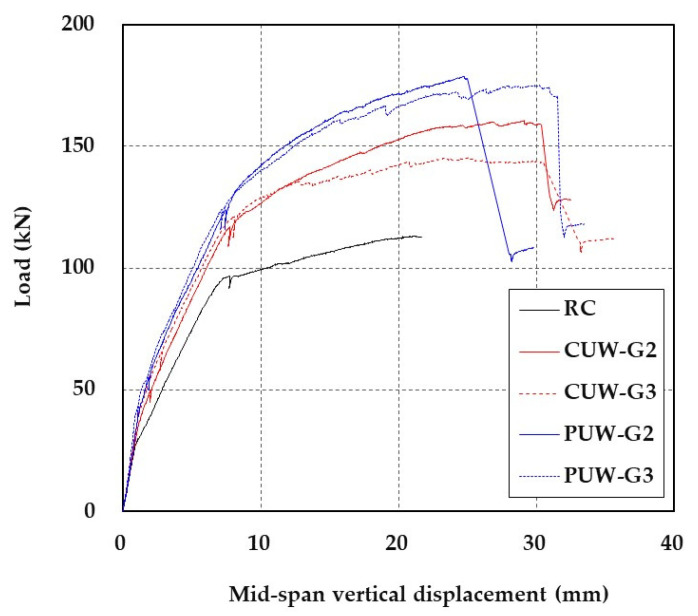
Load-displacement curve of RC slabs repaired with TRC grouting system.

Figure 12 shows the three-point flexural test set-up with a universal testing machine. Static loading was applied to the specimen with a loading head, and the mid-span vertical displacement was measured by a linear variable displacement transducer (LVDT). In Figure 13, the load versus displacement behavior of RC slabs repaired with the TRC grouting system is plotted, and the behavior is compared to that of the control specimen (RC). Furthermore, the test results for all sets of specimens are summarized in Table 8.

The RC slabs repaired with the TRC grouting system show a linear load-displacement behavior until concrete cracking occurs. After the concrete cracking, the stiffness of the repaired specimens is somewhat reduced, but the load could be further increased until the steel reinforcement yielded. The load-carrying capacity of the repaired specimen after the yield of steel reinforcement is due to the TRC grouting system. Overall, the ultimate load-carrying capacity of the PUW series specimens is at least 15.7% greater than that of CUW series specimens. Note that the thickness of the PUW series specimens is 20.0 mm greater than that of CUW series specimens. Therefore, a higher depth relative to the neutral axis increases the flexural capacity of the PUW series specimens. The load reaches its ultimate value of the repaired specimens, and an abrupt failure occurs. On the other hand, the load levels corresponding to the concrete cracking, steel yield, and failure of the RC specimens are at least 37.5%, 16.6%, and 21.7% smaller than those of the repaired specimens, respectively.

Table 8 also provides the results of flexural tests conducted in previous studies [25,28]. Note that the NA [25] and SG-1 [28] specimens have the same dimensions as the CUW series specimens, but are cast and cured in a dry condition with multi-purpose grout (G1). The load-carrying capacities of the NA and SG-1 specimens are almost identical to each other, but their average value is 4.3% smaller than the average values of the CUW series specimens. Therefore, if anti-washout grout is used, the influence of underwater installation of the TRC grouting system on the performance in concrete repair work is insignificant.

In Table 9, the test data are compared with analytical solutions. The analytical solutions were calculated by a procedure presented in the literature [19,28]. The steel yield load and ultimate load computed by the analytical procedure are, at most, 73% and 80% of the experimental results, respectively. Therefore, the load-carrying capacity of RC slabs repaired with the TRC grouting system can be estimated by the analytical procedure for design purposes with a safety margin.

### 3.5. Effect of Type of Grouting Material

As shown in Figure 13, the influence of the grouting materials on the load-displacement behavior of the slab specimens repaired with the TRC grouting system is not significant. For the CUW series specimens, the difference in steel yield load and ultimate load is, at most, 10%. On the other hand, for the PUW series specimens, the maximum difference in steel yield load and ultimate load is smaller than 4%.

**Figure 14:**
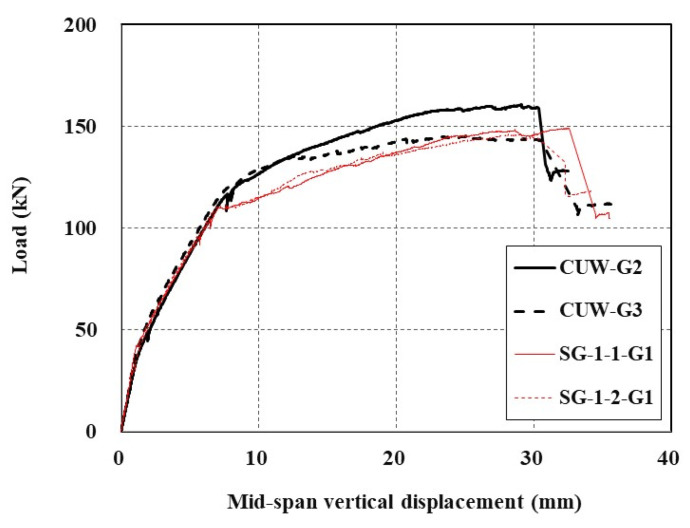
Load-displacement curves for CUW series and SG-1 series [28] specimens.

The load-displacement curves for the CUW series specimens and SG-1 series specimens [28] are plotted in Figure 14. The maximum load obtained for the CUW series specimen with G2 (underwater grout) is 8% greater than the average maximum load of the SG-1 series specimens [28] with G1 (multi-purpose grout). Note that the steel yield load of the CUW series specimens with underwater grouts is at least 15% greater than those of the SG-1 series specimens with the multi-purpose grout (G1).

### 3.6. Crack Pattern and Failure Mode

**Figure 15:**
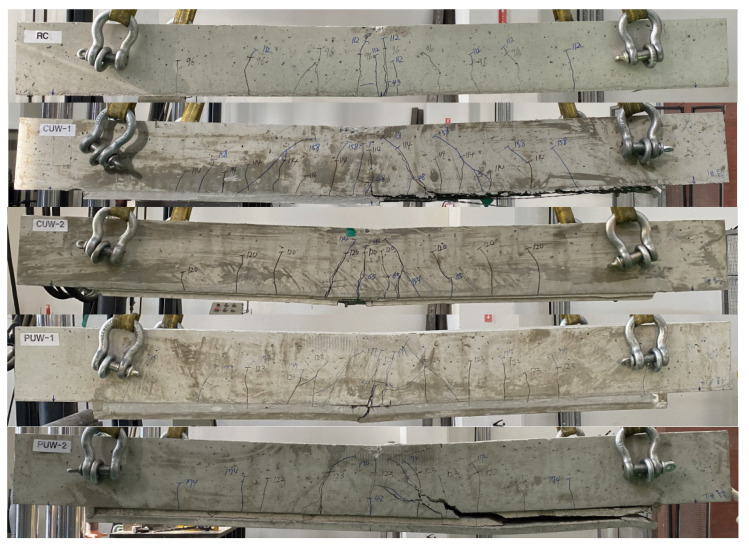
Crack patterns and failure modes of specimens (side view).

Figure 15 shows the crack patterns and failure modes of the specimens after the test. The RC specimen shows pure flexural cracks, and initially fails by the yielding of steel bars, followed by crushing of the top concrete. The RC slabs strengthened with the TRC grouting system (CUW-2 and PUW-1 specimens) also show flexural cracks, and then finally fail by a flexure failure mode. Furthermore, the CUW-2 and PUW-1 specimens fail by rupture of the textile. On the other hand, the CUW-1 and PUW-2 specimens show a diagonal shear failure mode, followed by debonding of the TRC grouting system from the RC slab at the ultimate stage.

## 4. Conclusions

(1) The bond strength of the repair material (grout) is greatly affected by the casting and curing conditions. When the multi-purpose grout is used as a repair material, the average bond strength of the specimens cast and cured in dry conditions is only 30% of the specimens cast and cured in underwater conditions. This result may be attributed to the fact that mixing water from the grout might be absorbed into the concrete substrate, and this adversely affects the bond strength of the concrete–grout interface. 

Non-dispersive, anti-washout grouts with normal and high compressive strengths (30 MPa and 50 MPa, respectively) were used for the bond test specimens, and cast in an underwater condition. The higher compressive strength of grout results in higher bond strength, but the maximum difference is 31.9%. Therefore, the non-dispersive, anti-washout grouts should be used for the underwater repair of concrete structures with the TRC grouting method.

(2) Four full-scale RC slab specimens were repaired with the TRC grouting method, two for each installation method. Regardless of the installation method, the load levels that cause concrete cracking, steel yield, and the failure of slab specimens repaired with the TRC grouting system are at least 37.5%, 16.6%, and 21.7% greater than those of the unrepaired specimen, respectively. The ultimate load-carrying capacity of specimens repaired with the TRC panel grouting system is at least 15.7% greater than that of specimens repaired with the CIP TRC grouting method. The test results further indicate that the influence of the grouting materials on the ultimate load-carrying capacity of the specimens repaired with the TRC grouting system is insignificant, and the maximum difference is, at most, 4%.

(3) In this study, the proposed method was evaluated by a trial fabrication and structural test for RC slab specimens. However, the number of specimens tested per each case was limited in terms of obtaining reliable results. Therefore, an additional test program with at least two specimens per case should be conducted in future study.

(4) Furthermore, several technical verifications should still be made for practical application. The total resources and time required for the proposed method should be compared with those of the conventional methods. A demonstration project should be conducted to evaluate the constructability of the proposed method for the repair and rehabilitation of deteriorated RC structures in an underwater environment. Successful development and validation of the proposed method will allow concrete structures in an underwater environment to be easily repaired.

## Figures and Tables

**Table 1:** Mix composition of concrete and mortar (unit: kg/m^3^).

Material	Cement ^1^	Water	Fly Ash	GGBS ^2^	Sand	Coarse Aggregate ^3^	Super- Plasticizer	Design Strength (MPa)
Concrete	263	167	56	56	828	934	2.63	27
Mortar	466	278	-	466	1024	-	3.00	30

^1^ Type I Portland cement specified in ASTM C150 [24]. ^2^ GGBS = granulated blast-furnace slag. ^3^ Maximum grain size = 25 mm.

**Table 2:** Design strength and manufacturer of cementitious grouts.

Grout ID	Grout Type	Design Strength (MPa)
G1	Multi-purpose repair	50
G2	Underwater repair	50
G3	Underwater repair	30

**Table 3:** Mixture composition of G1 (unit: kg/m^3^).

Cement ^1^	Sand	Water	Silica Fume	Super- Plasticizer	Expansion Agent	PVA Fibers ^2^
1055.0	1130.0	142.0	42.0	8.4	99.8	0.3%

^1^ Type I Portland cement specified in ASTM C150 [24]. ^2^ Polypropylene short fibers (length = 6.0 mm).

**Table 4:** Characteristic of bond test specimens.

Specimen ID	Specimen Type	Substrate Material	Repair Material	Interface	Casting Condition	No. of Specimen
STC	A	Concrete	-	-	Air	3
ST-1	B	Concrete	G1	Concrete–grout	Air	3
ST-2	B	Concrete	G1	Concrete–grout	Underwater	3
STU-1	B	Concrete	G2	Concrete–grout	Underwater	3
STU-2	B	Concrete	G3	Concrete–grout	Underwater	3
STP	B	Mortar	G1	Mortar–grout	Underwater	3
STPU-1	B	Mortar	G2	Mortar–grout	Underwater	3
STPU-2	B	Mortar	G3	Mortar–grout	Underwater	3

**Table 5:** Bond strength of specimens.

Specimen ID	Substrate Type	Grout Type	Interface	Bond Strength (MPa)	STD (MPa)	COV (%)
STC	Concrete	-	-	10.4	0.7	7.1
ST-1	Concrete	G1	Concrete–grout	1.5 ^1^	0.2	14.3
ST-2	Concrete	G1	Concrete–grout	5.0	0.2	3.4
STU-1	Concrete	G2	Concrete–grout	9.4 ^1^	2.0	21.2
STU-2	Concrete	G3	Concrete–grout	6.4	1.0	16.3
STP	Mortar	G1	Mortar–grout	6.5	1.0	15.1
STPU-1	Mortar	G2	Mortar–grout	4.8	1.0	21.6
STPU-2	Mortar	G3	Mortar–grout	10.2 ^1^	1.3	13.8

Note: ^1^ Average value of two tests. STD = standard deviation for bond strength. COV = coefficient of variation for bond strength.

**Table 6:** Characteristics of specimens for flexural test.

Specimen ID	Repair Method	Grout Type	No. of Textile	Formwork Type	Thickness of Specimen (mm)	No. of Specimens
RC	-	-	-	-	200	1
CUW-G2	CIP TRC	G2	1	Plywood	220	1
CUW-G3	CIP TRC	G3	1	Plywood	220	1
PUW-G2	TRC panel	G2	-	TRC panel	240	1
PUW-G3	TRC panel	G3	-	TRC panel	240	1
NA [25]	CIP TRC	G1	1	Plywood	220	1
SG-1 [28]	CIP TRC	G1	1	Plywood	220	2

**Table 7:** Material properties of carbon textile grid (suggested values by the manufacturer ^1^).

Fiber (Tex)	Resin	Surface Coating	Cross-Sectional Area of Textile ^2^ (mm^2^/m)	Tensile Strength (MPa)	Elastic Modulus (GPa)
3200	Epoxy	Sand-coated	85	3300	220

^1^ Q85/85-CCE-21-E4, Solidian-Kelteks, Karlovac, Croatia. ^2^ Cross-sectional area of yarn = 1.81 mm^2^.

**Table 8:** Results of flexural test for slab specimens.

Specimen ID	Steel Yield		Ultimate Stage		
	Load (kN)	Displ. (mm)	Load (kN)	Displ. (mm)	Load Gain (%)
RC	96.1	7.4	113.8	23.7	100.0
CUW-G2	115.2	7.5	160.5	28.8	141.1
CUW-G3	118.2	7.5	145.3	24.7	127.7
PUW-G2	126.9	7.8	178.7	24.4	157.1
PUW-G3	122.6	7.0	175.0	29.5	153.8
NA [25]	105.4	6.0	145.4	22.7	127.7
SG-1 [28] ^1^	101.7	6.2	147.6	29.1	129.7

Note: Disp. = mid-span vertical displacement. **^1^** Average value of two tests.

**Table 9:** Comparison of test data and analytical solutions.

Specimen ID	Analytical Solutions				Analytical Solutions/Test Data			
	Steel Yield		Ultimate Stage		Steel Yield		Ultimate Stage	
	Load (kN)	Disp. (mm)	Load (kN)	Disp. (mm)	Load (kN)	Disp. (mm)	Load (kN)	Disp. (mm)
CUW series	88.1	4.8	137.6	18.3	87%	77%	93%	63%
PUW series	90.9	4.8	141.8	16.5	73%	65%	80%	61%

Note: Disp. = mid-span vertical displacement.

## Data Availability

The data presented in this study are available on request from the corresponding author.
